# OncomiR-196 promotes an invasive phenotype in oral cancer through the NME4-JNK-TIMP1-MMP signaling pathway

**DOI:** 10.1186/1476-4598-13-218

**Published:** 2014-09-19

**Authors:** Ya-Ching Lu, Joseph T Chang, Chun-Ta Liao, Chung-Jan Kang, Shiang-Fu Huang, I-How Chen, Chi-Che Huang, Yu-Chen Huang, Wen-Ho Chen, Chi-Ying Tsai, Hung-Ming Wang, Tzu-Chen Yen, Guo-Rung You, Chang-Hsu Chiang, Ann-Joy Cheng

**Affiliations:** Department of Medical Biotechnology, College of Medicine, Chang Gung University, 259 Wen-Hwa 1st Road, Taoyuan, 333 Taiwan; Department of Radiation Oncology, Chang Gung Memorial Hospital, Taoyuan, Taiwan; Department of Otorhinolaryngology, Head Neck Surgery, Chang Gung Memorial Hospital, Taoyuan, 333 Taiwan; Department of Oral and maxillofacial Surgery, Chang Gung Memorial Hospital, Taoyuan, 333 Taiwan; Department of Medical Oncology, Chang Gung Memorial Hospital, Taoyuan, 333 Taiwan; Molecular Imaging Center and Department of Nuclear Medicine, Chang Gung Memorial Hospital, Taoyuan, 333 Taiwan

**Keywords:** miR-196, Cell invasion, NME4, JNK signaling, Oral cancer, Clinical association

## Abstract

**Background:**

MicroRNA-196 (miR-196), which is highly up-regulated in oral cancer cells, has been reported to be aberrantly expressed in several cancers; however, the significance of miR-196 in oral cancer has not yet been addressed.

**Methods:**

Cellular functions in response to miR-196 modulation were examined, including cell growth, migration, invasion and radio/chemosensitivity. Algorithm-based studies were used to identify the regulatory target of miR-196. The miR-196 target gene and downstream molecular mechanisms were confirmed by RT-qPCR, western blot, luciferase reporter and confocal microscopy analyses. miR-196 expression was determined in paired cancer and adjacent normal tissues from oral cancer patients.

**Results:**

Both miR-196a and miR-196b were highly over-expressed in the cancer tissue and correlated with lymph node metastasis (*P* = 0.001 and *P* = 0.006, respectively). Functionally, miR-196 actively promoted cell migration and invasion without affecting cell growth. Mechanistically, miR-196 performed it's their function by inhibiting NME4 expression and further activating p-JNK, suppressing TIMP1, and augmenting MMP1/9.

**Conclusion:**

miR-196 contributes to oral cancer by promoting cell migration and invasion. Clinically, miR-196a/b was significantly over-expressed in the cancer tissues and correlated with lymph node metastasis. Thus, our findings provide new knowledge of the underlying mechanism of cancer metastasis. miR-196 may serve as a promising marker for better oral cancer management.

**Electronic supplementary material:**

The online version of this article (doi:10.1186/1476-4598-13-218) contains supplementary material, which is available to authorized users.

## Novelty and impact statement

In this study, we elucidated the significance of miR-196 in oral cancer. miR-196 promotes cell migration and invasion. Mechanistically, miR-196 exerts these functions by targeting to the NME4 molecule and regulating the downstream JNK-TIMP1-MMP signaling pathway. In addition, both miR-196a and miR-196b were remarkably up-regulated in oral cancer tissues and correlated with lymph node metastasis. Thus, miR-196 could be a promising marker for better management of oral cancer.

## Introduction

Oral cancer is one of the most prevalent cancers worldwide [1]. Despite improvements in diagnosis and treatment in recent decades, the survival rate for oral cancer has not significantly changed due to the development of distant metastases and therapeutic resistance [2–4]. It is essential to thoroughly investigate the pathogenesis of this disease to provide fundamental knowledge for future clinical applications.

MicroRNAs (miRNAs) constitute an abundant class of small, non-coding RNA molecules that regulate gene expression by targeting mRNAs to induce translational repression or mRNA degradation [5]. Increasing evidence indicates that miRNAs contribute to the development of cancer by negatively regulating target gene expression, and therefore they can function as tumor suppressors or oncogenes [6, 7]. Recently, miRNA screening in several types of cancer has identified unique expression profiles associated with specific tissues or clinical features, including head and neck cancer [8, 9]. To improve the understanding of the role of miRNAs in oral cancer, we previously performed global miRNA profiling of normal keratinocyte and cancer cell lines. We discovered 23 miRNAs with significantly altered expression in cancer cells, including miR-196 [10]. miR-196 has been reported to be aberrantly expressed in various malignancies, including melanoma, leukemia, and glioblastoma [11–21]. However, the underlying mechanism by which these molecules cause malignancy remains unclear.

In the present study, we characterized the function of miR-196 and elucidated it’s molecular mechanism in oral cancer. We found that the miR-196 family positively regulated cell invasion and migration, and had no effect on cell growth. Mechanistically, miR-196 exerted their effects by directly targeting and inhibiting non-metastatic cells 4 (NME4) protein expression to regulate the JNK-TIMP1-matrix metalloproteinase (MMP) signaling pathway. We revealed that both miR-196a and miR-196b were highly over-expressed in the cancer tissues of patients with oral cancer, demonstrating the clinical significance of these molecules during cancer progression.

## Materials, subjects, and methods

### Cells and cell lines

Four oral cancer cell lines (OECM1, SAS, CGHNC8, and CGHNC9) and two normal keratinocyte cell lines (CGHNK2 and CGHNK4) were used [10]. CGHNK2 and CGHNK4 cells are HPV-immortalized lines of normal keratinocytes that were described previously [10]. The immortalized normal keratinocyte cells were maintained in KSFM medium (Life Technologies, Inc., Gibco BRL, Rockville, MD, USA). The cancer cell lines were grown in 100% DMEM or RPMI 1640 medium containing 10% fetal bovine serum (Life Technologies, Inc.). All cells were cultured at 37°C in a humidified atmosphere with 5% CO_2_.

### Cloning and transfection of miR-196–specific plasmids and inhibitory antagomir oligonucleotides

All the oligonucleotides used in this study, including the specific stem-loop sequences of miR-196a, miR-196b, the inhibitory antagomir oligonucleotides (Anti-196a and Anti-196b), random sequence for antagomir control (RC) are listed in Additional file 1: Table S1. The stem-loop oligonucleotides were inserted into the multiple cloning site of the pcDNA 3.1(+) expression vector (Invitrogen, Carlsbad, CA, USA) to construct the miR-196 overexpression plasmids. To promote miR-196 expression, 3 μg of miR-196 plasmid was transfected into cells plated in 100-mm dishes. The miR-196a, miR-196b antagomir and the random sequence oligonucleotides for controls were purchased from TRI-I Biotech, Inc. (New Taipei City, Taiwan). To suppress miR-196 expression, 300 μM antagomir oligonucleotides were transfected into the cells. Transfection was performed using the Lipofectamine 2000™ reagent (Invitrogen) in OPTI-MEM medium (Invitrogen), and the cells were incubated at 37°C in a humidified atmosphere with 5% CO_2_ for 10 h, similarly as previously described [22]. Afterward, the medium was replaced with fresh complete medium, and the cells were continuously cultured.

### Cell migration assay

Cell migration was determined using an *in vitro* wound healing assay as previously described [23]. After transfection of the miR-196 overexpression plasmids or the antagomir oligonucleotides, 3.5 × 10^4^ cells were seeded in ibidi® culture inserts (ibidi LLC, Verona, WI, USA) on top of a 6-well plate. After 8 h of incubation, the culture inserts were detached to form a cell-free gap in the cell monolayer. After changing to culture medium containing 1% FCS, cell migration of toward the gap area was photographed every 6 h. All the experiments were performed at least three times independently and that typical results were shown. In each sample, the invasion ability was quantified by comparing the distance of the cell-free gap after normalization to the control group. The error bars shown in the relevant figures indicated the standard deviation of the quantification results in all experiments.

### Cell invasion assay

The invasive abilities of the cells were determined by culturing the cells on a polycarbonate membrane coated with Matrigel (Becton Dickinson Biosciences, Franklin Lakes, NJ, USA) in a Millicell invasion chamber (Millipore, Billerica, MA, USA) as previously described [23]. Briefly, Matrigel was first coated onto the membrane of the Millicell upper chamber for 12 h at 37°C. After transfection of the miR-196 overexpression plasmids or antagomir oligonucleotides, the cells were seeded in the upper chamber with 1% FBS medium. The lower chamber contained complete culture medium (containing 10% FBS) to trap invading cells. After incubation at 37°C, the cells that invaded through the Matrigel-coated membranes into the lower chamber were stained with crystal violet and photographed. All the experiments were performed at least three times independently and that typical results were shown. In each sample, the invasion ability was quantified by comparing the density of crystal violet dye after normalization to the control group. The error bars shown in the relevant figures indicated the standard deviation of the quantification results in all experiments.

### mRNA and miRNA analysis by reverse transcription-quantitative PCR (RT-qPCR)

Total RNA was isolated from cells using TRIzol reagent (Gibco BRL). For mRNA determination, RT was performed as previously described [24]. For miRNA determination, the revere transcription was performed as previously described [10] using miR-196–specific stem-loop RT primers and assay kits (ABI, Forest City, CA, USA) according to the manufacturer’s suggested protocol. The PCR primers used for target genes are list in Additional file 1: Table S1. The results of real-time PCR, recorded as threshold cycle numbers, were normalized against an internal control (U6 RNA for miRNAs or GAPDH for mRNA). The comparative threshold cycle (ΔΔCt) method was used to determine the gene expression. All the experiments were performed duplicate for at least three times. The error bars shown in the relevant figures indicated the standard deviation of the quantification results in all experiments.

### Protein extraction and western blot analysis

Protein extraction and western blot analysis were performed as previously described [25]. Briefly, cellular proteins were extracted using lysis buffer by incubating the cells on ice for 30 min. Samples were centrifuged at 14,000 *g* for 30 min, and the supernatant was collected. The protein samples were boiled at 95°C for 5 min, separated by electrophoresis on 10% polyacrylamide gels containing 0.1% SDS, and transferred to nitrocellulose membranes. The membranes were incubated with primary followed by horseradish peroxidase-conjugated secondary antibodies. The primary antibodies used in this study are listed in Additional file 1: Table S2. The membranes were developed using an ECL developing solution (Millipore) followed by autoradiography. All the experiments were performed at least three times independently and that typical results were shown. In each sample, the protein expression shown in each band was quantified after normalization to the GAPDH expression level. The error bars shown in the relevant figures indicated the standard deviation of the quantification results in all experiments.

### Luciferase reporter assay for the NME4 3′-UTR

The pMIR-REPORT firefly luciferase vector plasmid (pMIR, Ambion, Grand Island, NY, USA) was used. The 3′-UTR region of wide-type NME4 was amplified by PCR and cloned downstream of the luciferase vector (p-UTR-WT). A mutant sequence was also cloned as a validation plasmid (p-UTR-mut). pMIR, p-UTR-WT, and p-UTR-mut was co-transfected with the miR-196–specific antagomirs or overexpression plasmids into OECM1 or SAS cells. The pRL-SV vector (Promega, Madison, Wisconsin, USA) containing *Renilla* luciferase was also transfected for each condition as a reference control. Firefly and *Renilla* luciferase activities were measured using the Dual-Luciferase Reporter Assay System (Promega) according to the manufacturer’s instructions [26]. All the experiments were performed triplicate for at least three times, and the similar results were obtained. The error bars shown in the relevant figures indicated the standard deviation of a triplicate experiment.

### Immunofluorescence staining and confocal microscopy

Immunofluorescence staining and confocal microscopy were performed as previously described [27]. Briefly, cells were seeded onto coverslips coated with poly-L-lysine and incubated overnight at 37°C. After washing, the cells were fixed with formaldehyde, permeabilized with a permeation buffer, and blocked with 1% FBS. After overnight incubation with primary antibodies, the coverslips were incubated with fluorescence-conjugated secondary antibodies (Molecular Probes, Invitrogen, Carlsbad, CA, USA). The coverslips were then mounted with mounting medium containing DAPI dye (Vector Laboratories, Burlingame, CA, USA), and the fluorescence was visualized using a confocal laser microscope (Leica TCS Sp2 MP).

### Patients and clinical association study and statistical analysis

This study was approved by the Institutional Review Broad of the Human Investigation Committee in Chang Gung Memorial Hospital. Written informed consent was obtained from all patients participating in this study. Fifty-four patients who visited Chang Gung Memorial Hospital (Taoyuan, Taiwan) were recruited for this study. The characteristics of these patients are summarized in Table 1. This study consisted of 5 (9%) females and 49 (91%) males. The mean age of the patients was 54.6 years old, with a median age of 53.0 years (range, 35–77 years). A total of 25 patients (46%) consumed alcohol, 30 patients (56%) smoked cigarettes, and 37 (68%) chewed betel quid. Cancer lesions were in oral tongue (n = 19, 35%), buccal mucosa (n = 17, 32%), other oral cavity sites (n = 17, 31%), or soft plate (n = 1, 1.9%). The surgically dissected cancer tissues and small pieces of adjacent normal counterpart were obtained before chemotherapy or radiotherapy. Twenty patients (37%) were receiving radical surgery, and 13 (24%) and 21 (39%) patients were receiving post-operative radiotherapy and concomitant chemoradiotherapy, respectively.

**Table 1:** **Characteristics 54 patients with oral cancer, and the association of miR-196a and miR-196b expressions with clinicopathological status**

	miR-196a		miR-196b	
**Characteristics (** ***n*** **)**	**low (** ***n*** **,%)**	**high (** ***n*** **,%)**	**low (** ***n*** **,%)**	**high (** ***n*** **,%)**
Sex				
Female (5)	0 (0)	5 (100)	1 (20)	4 (80)
Male (49)	12 (24)	37 (76)	18 (37)	31 (63)
*P value	0.210		0.455	
Age				
≤ 55 years old (30)	6 (20)	24 (80)	9 (30)	21 (70)
> 55 years old (24)	6 (25)	18 (75)	10 (42)	14 (58)
*P* value	0.661		0.372	
Alcohol drinking				
No (29)	6 (21)	23 (79)	10 (34)	19 (66)
Yes (25)	6 (24)	19 (76)	9 (36)	16 (64)
*P* value	0.770		0.907	
Cigarette smoking				
No (24)	3 (12)	21 (88)	7 (29)	17 (71)
Yes (30)	9 (30)	21 (70)	12 (40)	18 (60)
*P* value	0.124		0.407	
Betel quid chewing				
No (17)	3 (18)	14 (82)	7 (41)	10 (59)
Yes (37)	9 (24)	28 (76)	12 (32)	25 (68)
*P* value	0.584		0.532	
Pathologic T-status				
T1-T2 (25)	6 (24)	19 (76)	9 (36)	16 (64)
T3-T4 (29)	6 (21)	23 (79)	10 (34)	19 (66)
*P* value	0.770		0.907	
Pathologic N-status				
pN0 (32)	12 (37)	20 (63)	16 (50)	16 (50)
pN + (22)	0 (0)	22 (100)	3 (14)	19 (86)
*P value	0.001		0.006	
Pathologic stage				
I - II (19)	6 (32)	13 (68)	8 (42)	11 (58)
III - IV (35)	6 (17)	29 (83)	11 (31)	24 (69)
*P* value	0.223		0.433	
Differentiation				
Well (15)	5 (33)	10 (67)	6 (40)	9 (60)
Moderate (35)	7 (20)	28 (80)	13 (37)	22 (63)
Poor (4)	0 (0)	4 (100)	0 (0)	4 (100)
^*#*^ *P* value	0.137		0.280	
Total	12 (22)	42 (78)	19 (35)	35 (65)

*Fisher’s exact test was used.

^#^Liner-by-linear association test was used.

For each tissue, total RNA was extracted and subjected to miR-196a and miR-196b analyses as described previously. To define the relative levels of miR-196 in the clinical samples, the expression level of each tumor sample was normalized to an internal control (U6 RNA) and compared with that of normal tissue from the same patient. The cutoff points were determined after calculating the receiver operating characteristic (ROC) curve for best fit of sensitivity and specificity. Expression levels greater than 15-fold for miR-196a and 7-fold for miR-196b in tumor tissues compared to the expression in normal tissue were defined as ‘high.’ Level. The Pearson chi-square test was used to examine the association of miR-196 expression with clinicopathologic features, including TNM stage. Survival curves were calculated by the Kaplan-Meier method with a log-rank test. All *P* values were two-sided, and the significance level was set at *P* < 0.05.

## Results

### Both miR-196a and miR-196b promote cell migration and invasion without affecting cell growth

**Figure 1:**
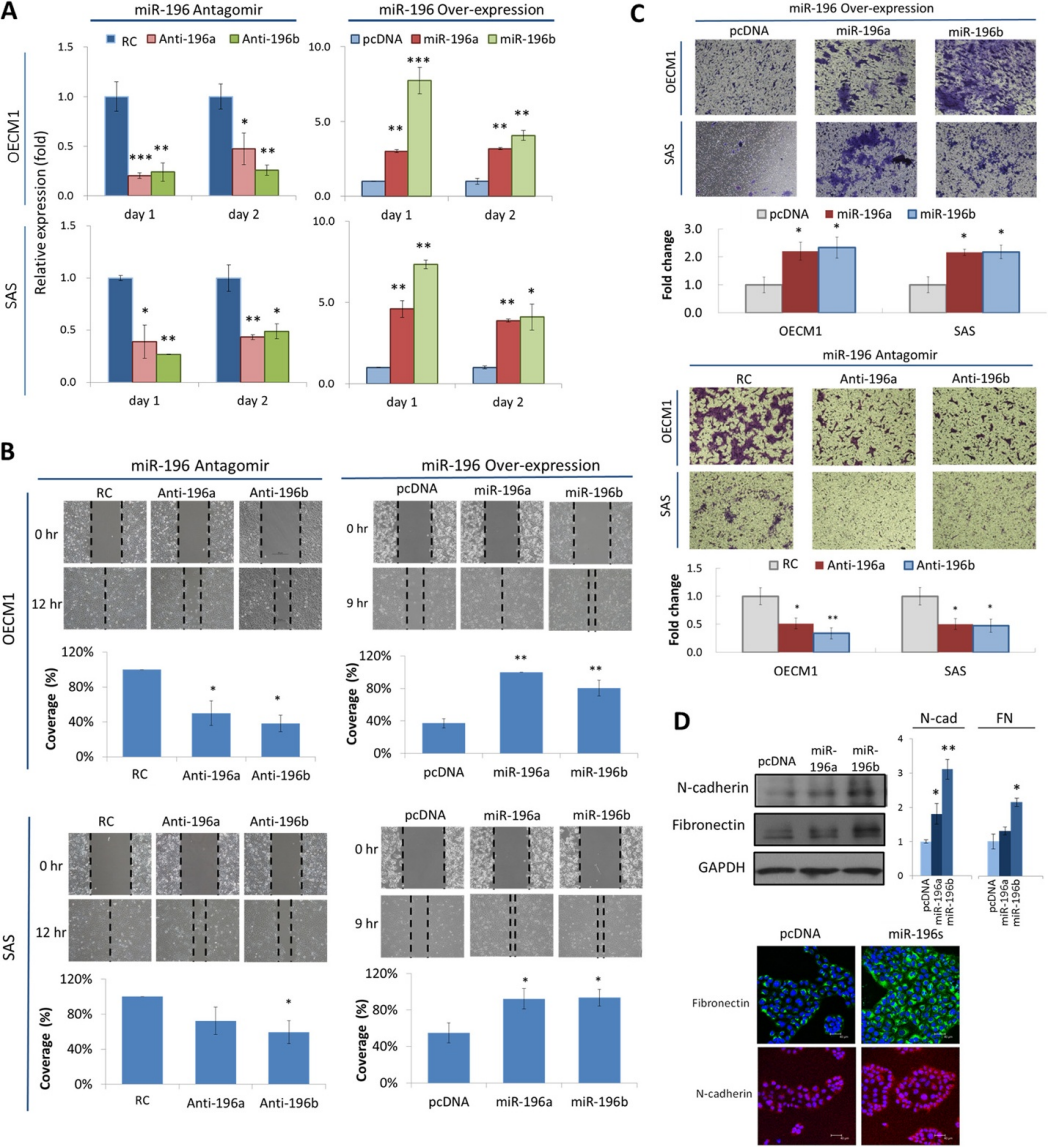
**Both miR-196a and miR-196b promote migration and invasion without affecting cell growth. (A)** Modulation of miR-196a and miR-196b expression after transfection of specific antagomirs or over-expression plasmids into OECM1 and SAS cells. These included miR-196 antagomirs (Anti-196a, Anti-196b), the random sequence for antigomir control (RC), the over-expression plasmids (miR-196a, miR-196b), or the expression vector (pcDNA). After 1 days of transfection, cells were harvested, and the levels of miR-196a and miR-196b were determined as described in the Methods section. **(B)** Effect of miR-196 modulation on cell growth and colony formation. After transfection of miR-196–specific antagomirs (Anti-196a, Anti-196b) or random oligonucleotides (RC) for 1 day, 1 × 10^6^ cells were re-seeded in 100 mm plates with for 3 days to allow cell growth, or 1 × 10^5^ cells were seeded for 7 days to allow colony formation. The number of colonies was determined. **(C)** Cells after modulating miR-196 level were applied to an *in vitro* wound healing migration assay as described in the Methods section. Cell migration toward the gap was observed, photographed, and quantified at the indicated times. **(D)** Effects of miR-196 modulation on cell invasion. After the transfection of miR-196 overexpression plasmids or the antagomir oligonucleotides, cells were subjected to a Matrigel invasion assay as described in the Methods section. The cells that invaded through the Matrigel-coated membranes to the lower chamber were stained, photographed, and quantified at the indicated times. (*p < 0.05 , **p < 0.01, ***p < 0.005, t-test).

To determine the carcinogenic functions of miR-196a and miR-196b, *in vitro* loss-of-function experiments using antagomir oligonucleotides and gain-of-function experiments using miRNA plasmid transfections were performed. The results indicated that the antagomirs against miR-196a and miR-196b substantially inhibited their expression by 79–80 and 62–73%, respectively, in OECM1 and SAS cells after 1 day (Figure 1A). Plasmid transfection upregulated miR-196a and miR-196b levels by 2.8–5.7- and 4.6–7.1-fold, respectively, in OECM1 and SAS cells after 1 day (Figure 1A). The potential effect of miR-196a or miR-196b on cell growth was examined in OECM1 and SAS cells. As shown in Additional file 2: Figure S1, silencing of miR-196a or miR-196b had no effect on cell proliferation. Similarly, over-expression of miR-196a or miR-196b has no significant effect on cell colony growth. These results suggested that miR-196 has minimal effect on growth regulation.

The potential effect of miR-196 on and chemo/radio-sensitivity was also examined using a clonogenic survival assay. Silencing of miR-196a or miR-196b had no effect on cell survival in response to cisplatin treatment (Additional file 2: Figure S2A). However, miR-196a and miR-196b had differential effects on radiosensitivity. Whereas miR-196b depletion had no effect, both cell lines were significantly more sensitive to radiation after miR-196a silencing (Additional file 2: Figure S2B). This result suggests that miR-196a, but not miR-196b, protects cells against radiation damage.

Cell migration and invasion were next analyzed using *in vitro* wound healing and Matrigel invasion assays. As shown in Figure 1B, miR-196 silencing resulted in slower migration toward the gap area in both OECM1 and SAS cells, with reductions of 28-50% and 41-62%, respectively, for miR-196a and miR-196b at 12 hours. Consistently, miR-196 over-expression significantly enhanced cell migration in both cell lines, with 1.9-2.7- and 1.7-2.2-fold increases, respectively, for miR-196a and miR-196b at 9 hours. Similar effects were also observed in cell invasion ability (Figure 1C). Depletion of miR-196a or miR-196b dramatically reduced the invading cells by 40-50% in both OECM1 and SAS cells. Consistently, miR-196a or miR-196b over-expression significantly enhanced cell invasion by 2.2-fold in both cell lines. Supporting these cellular findings, the expressions of cell adhesion molecules N-cadherin and fibronectin were up-regulated in the miR-196 over-expressing cells (Figure 1D). Collectively, miR-196a and miR-196b promote migration and invasion in oral cancer cells but exhibit minimal effects on cell growth.

### NME4 is a direct regulatory target of miR-196

**Figure 2:**
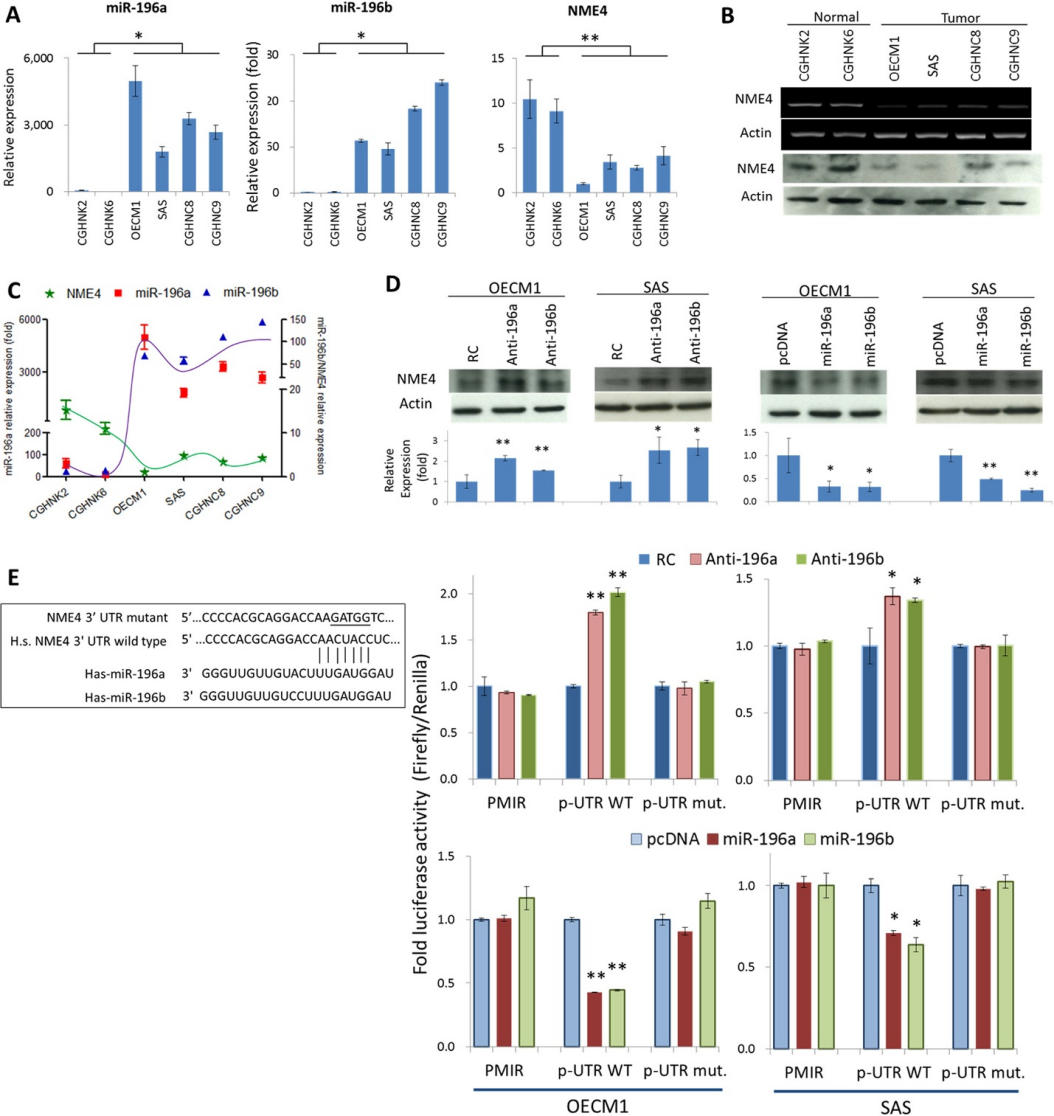
**NME4 is a direct regulatory target of miR-196. (A)** Differential expression of miR-196a/b and NME4 mRNA in two immortalized normal keratinocyte and four oral cancer cell lines, as determined by RT-qPCR. Relative levels of miR-196a/b were determined after normalization to the U6 RNA level (internal control) for each sample. The NME4 RNA level was normalized to actin for each sample. **(B)** Differential expression of NME4 mRNA and protein in two immortalized normal keratinocyte and four oral cancer cell lines, as determined by RT-PCR and western blotting. Actin expression was also determined as an internal control. **(C)** Inverse correlation between NME4 and miR-196a/b expression which determined by RT-qPCR. **(D)** Effect of miR-196 modulation on NME4 mRNA expression. OECM1 and SAS cells transfected with miR-196a/b–specific antagomirs or overexpression plasmids were harvested. Total protein was extracted and subjected to western blot analysis of NME4 expression. The relative expression was determined after normalization to actin as an internal control. **(E)** The mature sequences of miR-196a and miR-196b, as well as the 3′-UTR sequence of the human NME4 gene, are shown. The mutant sequence of the 3′-UTR region of NEM4 was designed. Luciferase reporter assay was performed to determine whether NME4 is a direct miR-196a/b target gene. Cells transfected with miR-196–specific antagomirs or overexpression plasmids were co-transfected with pMIR, p-UTR-WT, or p-UTR-mut. *Renilla* luciferase was also transfected as a reference control for each condition. Firefly and *Renilla* luciferase activities were measured using the dual-luciferase reporter assay. (*p < 0.05 , **p < 0.01, ***p < 0.005, t-test).

To identify the potential target of miR-196, computational prediction software, including PicTar, miRanda, and TargetScan, was used. These programs identified common candidates for both miR-196a and miR-196b (Additional file 2: Figure S3). The expression of these genes was further confirmed by RT-qPCR in response to miR-196 modulation. Four genes (ABCB9, HOXA5, MGATA4, and NME4) were upregulated by more than 20% after miR-196 depletion, whereas three genes (HOXB6, SMCR8, and NME4) were downregulated by more than 20% after miR-196 overexpression (Additional file 2: Figure S3). Of these genes, only the expression of NME4 changed consistently in both confirmation studies. Therefore, NEM4 is a potential miR-196 regulatory target.To determine the association of miR-196 and NME4, the expression levels of these molecules were examined in two lines of normal keratinocytes and four oral cancer cell lines. As shown in Figure 2A, miR-196 was significantly up-regulated in all cancer cell lines compared to those in normal cells, with 92- and 71-fold higher in average for miR-196a and miR-196b respectively. By contrast, NME4 expression was reduced in all cancer cell lines at both mRNA (3.9 fold) and protein levels (2.3 fold) (Figures 2B). This reverse correlation between these molecules further suggests that NME4 is a regulatory target of miR-196 (Figures 2C).To further examine whether NME4 is a down-stream target of miR-196, the potential effect of NME4 protein expression was determined in response to miR-196 modulation. As shown in the Figure 2D, NME4 levels were elevated (1.4–2.9-fold) or reduced (52–78%) upon miR-196 silencing or over-expression. To validate the regulatory target of NME4, a luciferase reporter assay was performed. Reporter plasmids that carry human NME4-3’UTR wild-type sequence (p-UTR-WT) and mutant sequence (p-UTR-mut) (Figure 2E) were co-transfected with either miR-196 antagomirs or expression plasmids. Silencing miR-196a or miR-196b increased NME4 wild-type UTR reporter activity in both OECM1 (1.7–2.0-folds) and SAS (1.3-fold) cells but had no effect on mutant UTR or empty vector reporter activity. Consistently, over-expression of miR-196a or miR-196b reduced NME4 wild-type UTR reporter activity both cell lines (by 57% in OECM1 cells and 29-36% in SAS cells). However, these miR-196 modulations exhibited minimal effects on mutant UTR reporter activity (Figure 2E). Taken together, these results suggest that NME4 is a down-stream regulatory target of miR-196.

### NME4 suppressed the effects of miR-196 on cell migration and invasion

**Figure 3:**
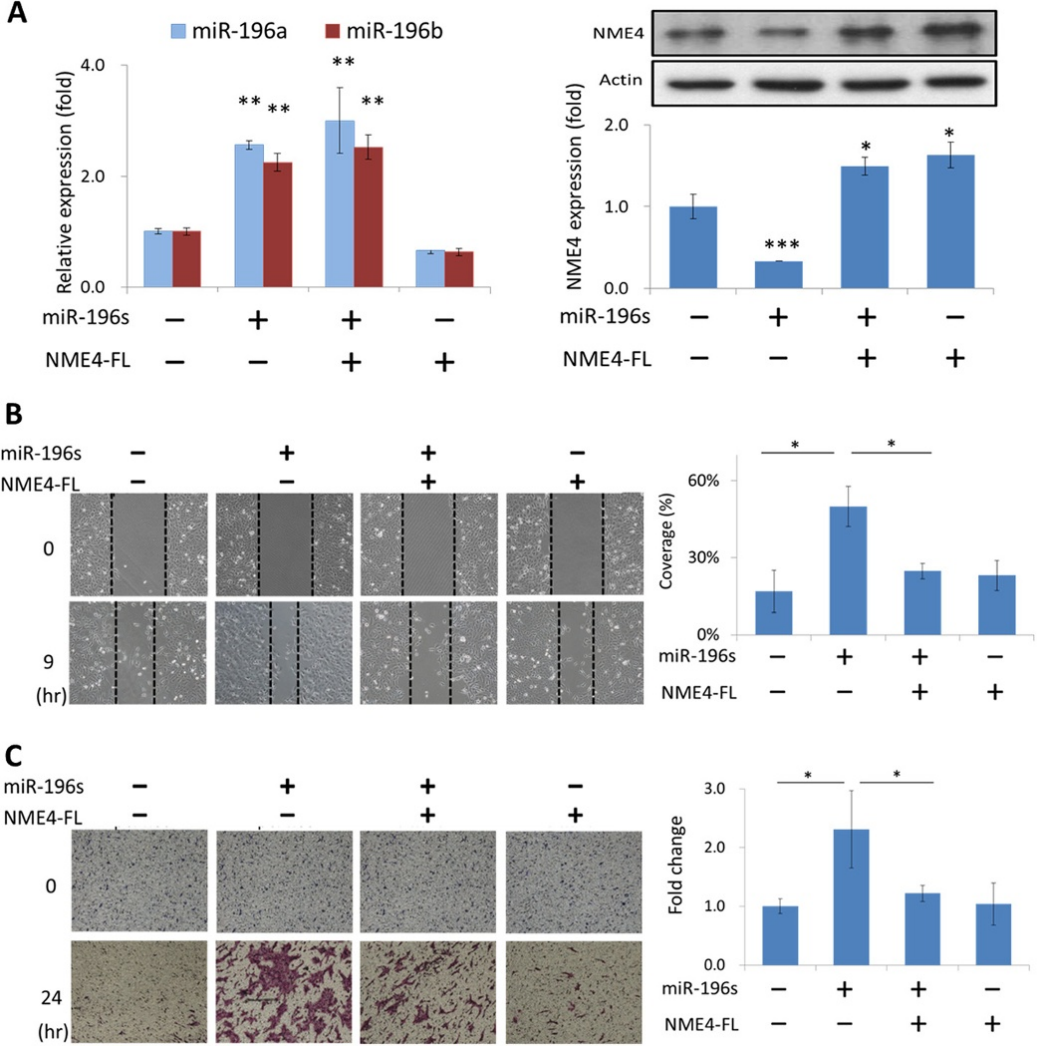
**NME4 suppressed the effect of miR-196s on cell migration and invasion. (A)** Verification of the expression status of miR-196 s and NME4 after transfection of the miR-196 overexpression or NME4 plasmids. After the transfection of miR-196 and/or NME4 plasmids into OECM1 cells, cell invasion **(B)** and migration **(C)** were examined as described in the Methods section. The empty vectors (pcDNA for miR-196 and pCIneo for NME4) were added if necessary to make total DNA amount being equally used in all experiments. (*p < 0.05, **p < 0.01, ***p < 0.005, t-test).

To investigate whether the enhancement of cell migration and invasion by miR-196 occurred via the suppression of NME4, these cellular effects were analyzed upon exogenous expression of NME4 in miR-196–overexpressing cells. After verifying the expression status of miR-196 and NME4 upon specific plasmid transfection (Figure 3A), cell invasion and migration were examined (Figures 3B-C). MiR-196 transfection significantly promoted cell invasion (2.3-fold) and migration (2.1-fold). However, cell invasion (Figures 3B) and migration (Figures 3C) were inhibited by 39 and 43%, respectively, upon exogenous NME4 expression. Transfection of NME4 alone had no effect on cell invasion or migration. Hence, the effect of miR-196 on cell migration and invasion is NME4-dependent.

### Cellular function of miR-196 occurs through the NME4-JNK-TIMP1-MMP1/9 molecular pathway

**Figure 4:**
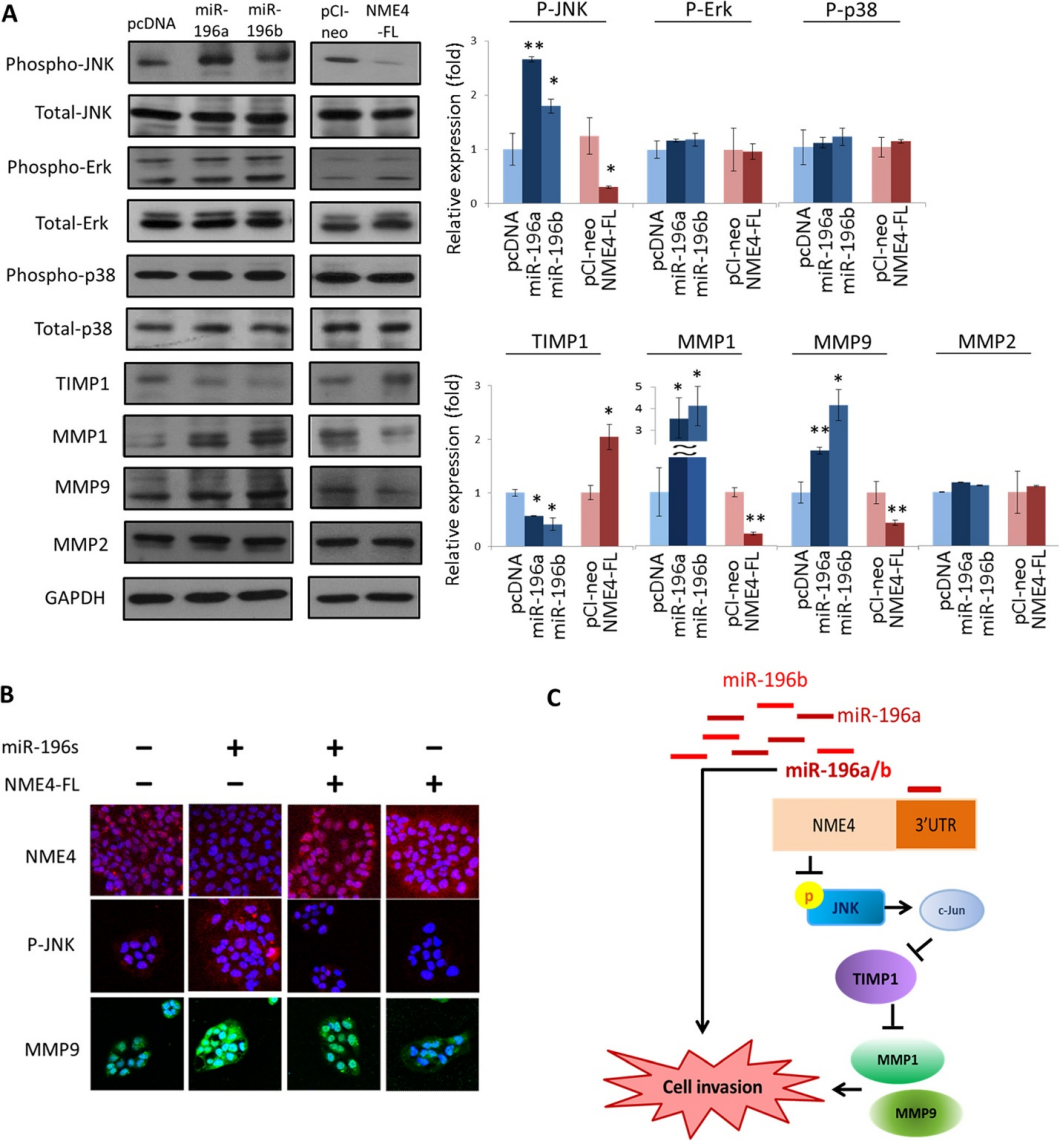
**Cellular effect of miR-196 is exerted via the NME4-JNK-TIMP1-MMP1/9 molecular pathway in OECM1 cells. (A)** Effects of miR-196 or NME4 plasmid transfection on MAPK, TIMP1 and MMP protein expression in OECM1 cells. Protein expression was determined by western blot analysis, and the density of each band was quantified. **(B)** Effect of miR-196 or NME4 plasmid transfection on p-JNK and MMP9 expression in OECM1 cells. Protein expression was determined by immunofluorescence staining and confocal microscopy. **(C)** A hypothetical model illustrating the miR-196-NME4-JNK-TIMP1-MMP1/9 molecular signaling pathway in oral cancer. (*p < 0.05, **p < 0.01, ***p < 0.005, t-test).

The mitogen-activated protein kinase (MAPK) pathway has been well characterized and demonstrated to play an important role in cell mobility [28, 29]. We investigated whether the effect of the miR-196–NME4 axis on cellular functions was regulated by MAPK molecules. Possible alterations in the phosphorylation status on three MAPK molecules, JNK, Erk, and p38, were examined by immunoblotting upon the modulation of miR-196 or NME4 expression via plasmid transfection. As shown in Figure 4A, miR-196 and NME4 had minimal effects on phospho-Erk (p-Erk) and phospho-p38 (p-p38) levels. However, phospho-JNK (p-JNK) levels were significantly increased by 2.6- and 1.8-fold by miR-196a and miR-196b modulation, respectively, whereas p-JNK levels were reduced to 0.7-fold of control levels by NME4 modulation. These results suggest that the miR-196–NME4 axis stimulates JNK phosphorylation.

The MMP family of proteins (MMP1, MMP2, and MMP9) and their tissue inhibitor TIMP1, which can promote or inhibit the digestion of the extracellular matrix [30, 31] were also examined (Figure 4A). Consistently, exogenous expression of miR-196a or miR-196b suppressed TIMP1 expression and enhanced MMP1 and MMP9 expression. Consistently, exogenous NME4 elevated TIMP1 expression but suppressed MMP1 and MMP9 expression. These results suggest that miR-196 promotes cell invasion through the NME4-JNK pathway, leading to the suppression of TIMP1 activity and elevation of MMP1/9 activity.

To determine whether JNK phosphorylation affects MMP expression, the JNK inhibitor SP600125 was used. As shown in Additional file 2: Figure S5, the treatment with SP600125 (0–50 μM) suppressed phospho-c-Jun (p-c-Jun) expression with a concomitant increase in TIMP1 expression and decrease in MMP1/9 expression. These concentration-dependent alterations suggest that TIMP1 and MMP1/9 are downstream targets of p-JNK and p-c-Jun.

To validate the regulation of the miR-196-NME4-pJNK-TIMP-MMP pathway, immunofluorescence staining and confocal microscopy were performed. As shown in Figure 4B, exogenous miR-196 reduced NME4 expression and elevated p-JNK and MMP9 expression compared to the findings in the control. On the contrary, exogenous NME4 reduced p-JNK and MMP9 expression. Introduction of NME4 in miR-196–overexpressing cells reversed the effect of miR-196 on p-JNK and MMP9 expression. Furthermore, this molecular pathway was also confirmed in another oral cell line (SAS) (Additional file 2: Figure S6). Taken together, these results suggest that miR-196 exerts it's effect through the NME4-pJNK-TIMP1-MMP1/9 pathway (Figure 4C).

### High expression of miR-196a and miR-196b in cancer tissues correlates with the clinical N-stage

**Figure 5:**
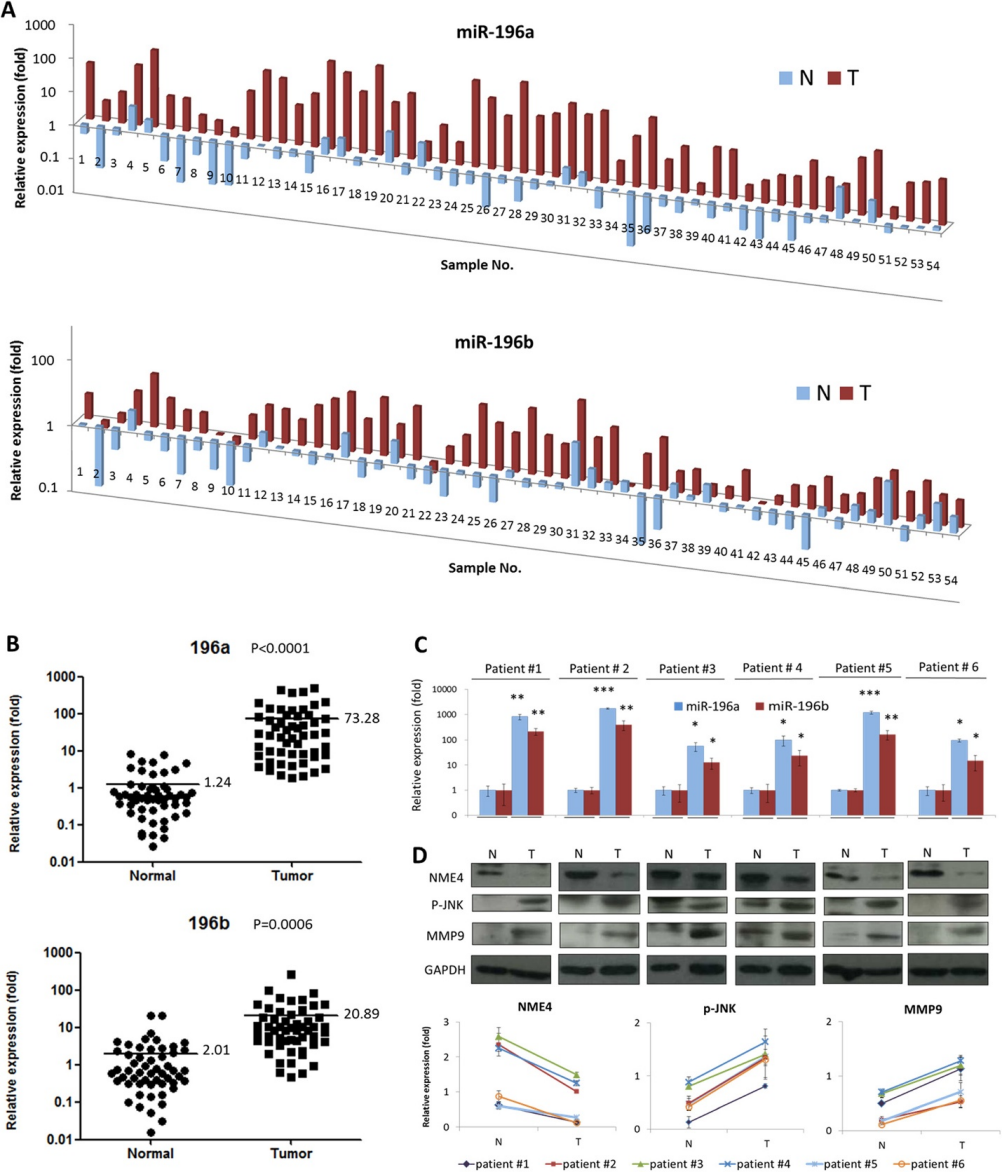
**High expression of miR-196a and miR-196b in cancer tissues.** A total of 54 paired cancer (T) and adjacent normal (N) tissues were obtained from patients with oral cancer. **(A)** For each sample, the expression levels of miR-196a and miR-196b were determined by RT-qPCR and normalized to U6 RNA as an internal control. **(B)** The relative expression of each sample is illustrated. The scatter dot plot shows the relative levels of miR-196a and miR-196b in the cancer and normal tissues from the patients. **(C-D)** Six paired of normal and oral cancer tissues were examined. **(C)** The expression level of miR-196a and miR-196b in the pairs of sample were determined by RT-qPCR based method. **(D)** The protein expressions of NME4, p-JNK and MMP9 were determined by western blot method, and showing GAPDH level as an internal control. Relative expressions of NME4, pJNK and MMP9 in each paired tissues. The density of each protein band was quantified after normalization to the GAPDH in each sample. The lines indicate the tendency of altered expressions of NME4, pJNK and MMP9 in each paired tissues. (*p < 0.05, **p < 0.01, ***p < 0.005, t-test).

To understand the clinical significance of miR-196, normal and cancerous tissues from 54 patients with oral cancer were obtained for analysis. For each tissue sample, the relative miRNA levels were determined, and the results are shown in Figure 5. Both miR-196a and miR-196b were substantially overexpressed in the cancer tissues. Compared to their normal counterparts, 96.3 (52 of 54) and 88.6% (48 of 54) of the cancer tissues exhibited greater than 2-fold higher expression of miR-196a and miR-196b (Figure 5A), respectively. On average, miR-196a and miR-196b levels were elevated by 59.1- and 10.4-fold, respectively, in the cancer tissues (Figure 5B).

To determine miR-196 downstream regulatory mechanism *in vivo*, six paired normal and cancerous oral cancer tissues were examined. As expected, miR-196a and miR-196b were significantly over-expressed in all cancer tissues (Figure 5C). Consistent with these cellular findings, the NME4 target molecule was substantially suppressed, and an elevation of phosphorylated JNK and MMP9 protein expression was observed (Figures 5D). These results confirmed that the dysregulation pathway of miR-196-NME4-pJNK-MMP molecular axis occurring in oral cancer patients.

To determine the potential association between cancer status and miR-196 expression, Pearson’s chi-squared test was used for statistical analysis. The associations of miR-196 expression with cancer stage and pathological status are shown in Table 1. There was no significant correlation of miR-196 expression with the pathological T stage, overall stage, differentiation status, alcohol consumption, cigarette smoking, or betel quid chewing. However, a significant correlation was found between high miR-196 levels and the pathological N stage (*P* = 0.001 and *P* = 0.006 for miR-196a and miR-196b, respectively). These results indicate the clinical significance of the miR-196 molecules in oral cancer.

## Discussion

The dysregulation of miRNAs is associated with malignant transformation. Previously, miR-196 expression was shown to be altered in several cancers. Although some investigators have reported decreased miR-196 expression in cancers, others have observed increased miR-196 expression. For example, miR-196a and miR-196b are down-regulated (or function as tumor suppressors) in melanoma [11] and acute lymphoblastic leukemia [17, 19]. However, miR-196a and miR-196b over-expression has been observed in several malignant diseases, including cancers of the esophagus [21], pancreas [20], colorectum [12], glioblastoma [14], and several types of leukemia [13, 18, 32]. High miR-196a levels have also been associated with a poor prognosis in pancreatic cancer, glioblastoma, and oral squamous cell carcinoma. Furthermore, the polymorphism of pre-miR-196a2 gene was observed in several malignant diseases, including head and neck cancer, and has been associated with cancer susceptibility or prognosis [33–37]. These studies indicate that miR-196 dysregulation plays an important role in carcinogenesis. Consistent with other reports, we previously observed miR-196 overexpression in oral cancer cell lines [10]. In that study, we further identified miR-196 overexpression in the cancer tissues of approximately 90% of patients with oral cancer compared to their expression in normal tissue (Figure 5), and this overexpression was associated with an aggressive phenotype with lymph node invasion (Table 1). These results demonstrate the significance of miR-196 in the development of oral cancer.

There are limited reports on the role of miR-196 in cancer development. In a study of breast cancer, ectopic miR-196a expression suppressed cell invasion, but the expression level of miR-196 was not correlated with the clinical metastatic status [15]. This result is different from the findings in gastrointestinal cancers, in which miR-196 overexpression promotes cell migration and invasion in colorectal and gastric cancer cells [12, 38, 39]. In another study, transfection of miR-196a mimic oligonucleotides into esophagus cells revealed that miR-196a promoted cell proliferation and suppressed apoptosis [40]. Thus far, the function of miR-196 in cancer remains obscure. In this study, we determined the role of miR-196 in oral cancer using both silencing and overexpression approaches. We found that both miR-196a and miR-196b actively promoted cell migration and invasion (Figures 1B-C), which were supported by the altered expression of fibronectin and N-cadherin (Figures 1D). However, neither miR-196a nor miR-196b affected cell growth and colony formation (Additional file 2: Figure S1). Hence, the main function of the miR-196 family in oral cancer is the regulation of cell mobility and invasiveness.

Identification of the target molecules of these miRNAs is of high interest. Previously, several molecules were reported as regulatory targets of miR-196. HOX family members have been reported primarily, including HOX-B7 (miR-196a target) in melanoma [16], HOX-A (miR-196b target) in acute lymphoblastic leukemia [18], and HOX-C8 (miR-196 family target) in melanoma [11] and breast cancer [15]. In this study, four HOX family genes (HOXA5, HOXB6, HOXB7, HOXC8) were identified as targets of miR-196. However, none of these genes responded to miR-196 perturbation (Additional file 2: Figure S4). This difference may be due to the distinct tissue specificity among the various regulatory targets of miR-196. In this study, after clarifying the correlation between NME4 and miR-196 expression in cells (Figures 2A-C), assessing the response of NME4 to miR-196 modulation (Figures 2D), we identified NME4 as a direct target of miR-196a and miR-196b in oral cancer using a luciferase reporter assay (Figure 2E),

NME4, also named nm23-H4, is a member of the nm23 family [41]. The proteins in this family possess nucleoside diphosphate kinase activity, which is believed to be involved in DNA repair mechanisms [42]. Nm23 family proteins also contain the RGD domain, as they can bind to integrin, and this family has been postulated to be involved in cell adhesion and migration [43]. Thus far, only a few studies assessed the association of NME4 with cancer, but genomic aberration or altered gene expression has been observed for NME4 in several types of cancers [44–47]. Although the function of NME4 is unclear, it was reported that an nm23 family member, NEM1, is regulated by TP53 [48] and that it acts as a metastatic suppressor [49]. In this study, we also found that ectopic expression of NME4 has no significant effect on cell invasion and migration (Figures 3B-C), indicating that a certain level of NME4 protein is sufficient for maintaining cellular mobility. However, restoration of silenced NME4 suppressed these effects induced by miR-196 (Figures 3B-C), suggesting that NME4 participates in the miR-196 regulatory pathway by inhibiting these functions. Collectively, miR-196 plays an oncogenic role by degrading NME4, thus accelerating cell migration and invasion.

The downstream regulatory mechanism of the miR-196–NME4 interaction was further investigated. In examining three MAPK family molecules, we found that p-JNK, but not p-Erk or p-p38, responded to miR-196 expression and NME4 inhibition, whereas miR-196 and NME4 had minimal effects on the expression of MAPK proteins (Figure 4A). These results indicate that miR-196–NME4 signaling could result in JNK phosphorylation and activation. In addition, TIMP1 and MMP1/9 displayed opposite responses to miR-196 suppression and NME4 augmentation (Figure 4A). These results suggest that TIMP1 and MMP1/9 are the downstream regulatory molecules of the miR-196–NME4 signaling axis. Additionally, we found that p-JNK inhibition increased TIMP1 expression and decreased MMP1/9 expression (Additional file 2: Figure S5). Hence, TIMP1 and MMP1/9 could be regulated by JNK phosphorylation. Moreover, the role of the NME4-pJNK-TIMP1-MMP1/9 signaling pathway in miR-196 function was further demonstrated by immunofluorescence staining and confocal microscopy (Figure 4B). Furthermore, this molecular pathway was also confirmed in another oral cell line (SAS) (Additional file 2: Figure S6) and paired normal and cancerous oral cancer tissues (Figure 5D). Thus, miR-196 appear to fine-tune the invasion mechanism in oral cancer by inhibiting NME4, leading to the activation of p-JNK and MMP1/9 and suppression of TIMP1 (Figure 4C).

In conclusion, we clarified that miR-196 promotes invasive and migratory phenotypes in oral cancer. Mechanistically, miR-196 exerted its functions by targeting to NME4, leading to the regulation of downstream molecules, including activating p-JNK, suppressing TIMP1, and augmenting MMP1/9. Consistently, clinical studies have revealed that both miR-196a and miR-196b are remarkably up-regulated in cancer tissue and correlated with lymph node metastasis. Thus, our findings provide new knowledge of the underlying mechanism of cancer metastasis. miR-196 may serve as a promising marker for better oral cancer management.

## Electronic supplementary material

Additional file 1: Table S1: Names and sequences of oligonucleotides of miR-196 stem-loop, the specific antigomirs and RT-PCR primers. **Table S2.** The primary antibody used in this study. (DOCX 24 KB)

Additional file 2: Figure S1: Both miR-196a and miR-196b has minimal effect on cell growth and colony formation in OECM1 and SAS cells. (A) After inhibition of miR-196s by antagomirs, cells were re-seeded to allow cell growth, and cell numbers were measured. (B) Cells were transfected with miR-196s over-expression plasmid to increase miR-196s expression. Clonogenic assay were performed and the number of colonies was determined. **Figure S2.** Neither miR-196a nor miR-196b affected chemosensitivity, whereas only miR-196a protects cells against irradiation (A) Chemoresistance was determined by the relative number of surviving cells after treatment with serial concentrations (0–5 μg/ml) of cisplatin for 2 days. In each sample, cell survival was counted and compared to the number of surviving untreated cell. (B) Radioresistance was determined using a clonogenic survival assay after treatment with various doses (0–6 Gy) of radiation for 7 days. **Figure S3.** Bioinformatic prediction of the regulatory targets of miR-196s using PicTar, TargetScan, and miRanda computational prediction software. The shared genes predicted by the three different prediction methods are listed in the table. **Figure S4.** Validation of the predicted genes by RT-qPCR. After transfection of the miR-196 antagomirs or overexpression plasmids into OECM1 cells, the mRNA level of each gene was determined by RT-qPCR. Relative expression was determined after each gene was normalized to the internal control (Actin). **Figure S5.** Effect of the JNK phosphorylation inhibitor SP600125 on TIMP1 and MMP protein expression in OECM1 cells. Protein expression was determined by western blot. **Figure S6.** (A) Effects of miR-196s or NME4 plasmid transfection on p-JNK, MMP1 and MMP9 in SAS cells. (B) Effect of the JNK phosphorylation inhibitor SP600125 on MMP protein expression in SAS cells. Protein expression was determined by western blot. (PDF 1 MB)
