# Supplementary material for: OncomiR-196 promotes an invasive phenotype in oral cancer through the NME4-JNK-TIMP1-MMP signaling pathway
Source: Mol Cancer. 2014 Sep 19;13:218. doi: 10.1186/1476-4598-13-218 (PMC4176851; doi:10.1186/1476-4598-13-218)
Supplement: Supplementary file 1 — Additional file 1: Table S1: Names and sequences of oligonucleotides of miR-196 stem-loop, the specific antigomirs and RT-PCR primers. Table S2. The primary antibody used in this study. (DOCX 24 KB) [file 12943_2014_1420_MOESM1_ESM.docx]

**Supplementary Tables**

**Supplementary Table ST1:** Names and sequences of oligonucleotides of miR-196 stem-loop, the specific antigomirs and RT-PCR primers.

| **Name** | **Sequence (5’ to 3’)** |
| --- | --- |
| **Antigomirs** |  |
| Anti-miR-196a (Anti-196a) | 5’-CCC AAC AAC ATG AAA CTA CCT A-3’ |
| Anti-miR-196b (Anti-196b) | 5’-CCC AAC AAC AGG AAA CTA CCT A-3’ |
| Random control sequence (RC) | 5’-NNN NNN NNN NNN NNN NNN NNN N-3’ |
| **Stem loop sequences** |  |
| miR-196a | 5’-GTG AAT TAG GTA GTT TCA TGT TGT TGG GCC TGG GTT TCT GAA CAC AAC AAC ATT AAA CCA CCC GAT TCA C-3’ |
| miR-196b  **RT-qPCR primers**  ABCB9  CDYL  COL1A2  GATA6  HAND1  HOXA5  HOXB6  HOXB7  HOXC8  MGAT4A  SMCR8  NME4 | 5’-ACT GGT CGG TGA TTT AGG TAG TTT CCT GTT GTT GGG ATC CAC CTT TCT CTC GAC AGC ACG ACA CTG CCT TCA TTA CTT CAG TTG-3’  F: 5’-GAA CAC AGT CAA GGT CAC G-3’  R: 5’-ATG GCA CTG ATG GTC TCC-3’  F: 5’- AAT CCA GAG GTA ATG AGA G-3’  R: 5’- CAC GAA GTT TCT GAT AGC-3’  F: 5’- TTA GCA CCA CGG CAG CAG-3’  R: 5’- ACT CCT TGT GTC GCA GAG C-3’  F: 5’- TTC CTA CGC TTC GCA TCC-3’  R: 5’- GAG GTC AGT GAA CAG CAG-3’  F: 5’- TAA TAC ATT AAG AGA AAG AG-3’  R: 5’- TAG GGA AAT GGA GAT AGG-3’  F: 5’- AAG GTG ACT TTC TGA AAC TCC-3’  R: 5’- CGC TAT AAT GGC AAT AAA CAG G-3’  F: 5’- CCC TCC CCA AAG CCA CTG-3’  R: 5’- AAG ACA AGA CTA CAA ACA CAA CAC-3’  F: 5’- GCT CTG CCT CAC GGA AAG-3’  R: 5’- TTC TCC ATC CCT CAC TCT TC-3’  F: 5’- TGA GGA AGA GGA GAA AGA AGA G-3’  R: 5’- GCA AGT ATC TGA CAG GTA GTT AG-3’  F: 5’- ACT TCC TTC CTT ACT TTG TCT TG-3’  R: 5’- GCA TCC TTA CTT CCA TTT GTT TC-3’  F: 5’- TCA TTC TTA TTT CCG AGT TCT C-3’  R: 5’- GTC CTC CAC GAA GTT CAG-3’  F: 5’- ATC AGC AGG AAT GTC ATC-3’  R: 5’- GGG AAG TAA GTG GTT TGG-3’ |

**Supplementary Table ST2:** The primary antibody used in this study

| **Name** | **Source** |
| --- | --- |
| NME4 | SC-99071, Santa Cruz, CA |
| Fibronectin  N-cadherin  MAPK family  JNK | SC-18825, Santa Cruz, CA  BD-610921, BD Biosciences, Franklin Lakes, New Jersey  #9926 family antibody sampler kit, Cell Signaling, Danvers, MA |
| Erk | #9926 family antibody sampler kit, Cell Signaling, Danvers, MA |
| p38 | #9926 family antibody sampler kit, Cell Signaling, Danvers, MA |
| phospho-MAPK family |  |
| phsopho-JNK | #9910 family antibody sampler kit, Cell Signaling, Danvers, MA |
| phosphor-Erk | #9926 family antibody sampler kit, Cell Signaling, Danvers, MA |
| phosphor-p38 | #9926 family antibody sampler kit, Cell Signaling, Danvers, MA |
| Metalloproteinase  TIMP1  MMP1  MMP2  MMP9 | MS-608, NeoMarker, Seattle, WA  #RB-1536, Thermo Fisher Scientific, Fremont, CA  SC-10736, Santa Cruz, CA  SC-10737, Santa Cruz, CA |
