# Supplementary material for: OncomiR-196 promotes an invasive phenotype in oral cancer through the NME4-JNK-TIMP1-MMP signaling pathway
Source: Mol Cancer. 2014 Sep 19;13:218. doi: 10.1186/1476-4598-13-218 (PMC4176851; doi:10.1186/1476-4598-13-218)

Supplementary Figure S1

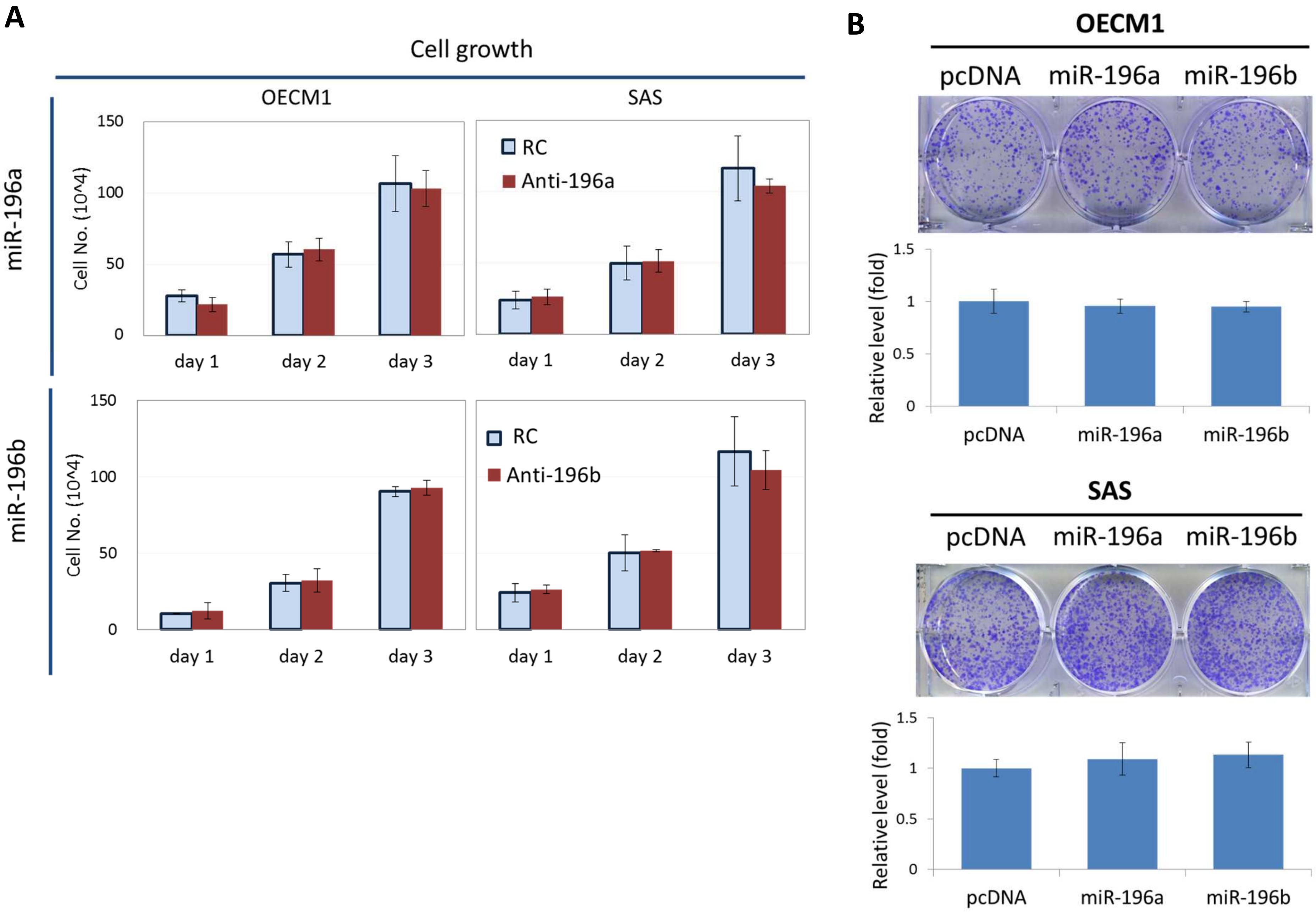

Supplementary Figure S2

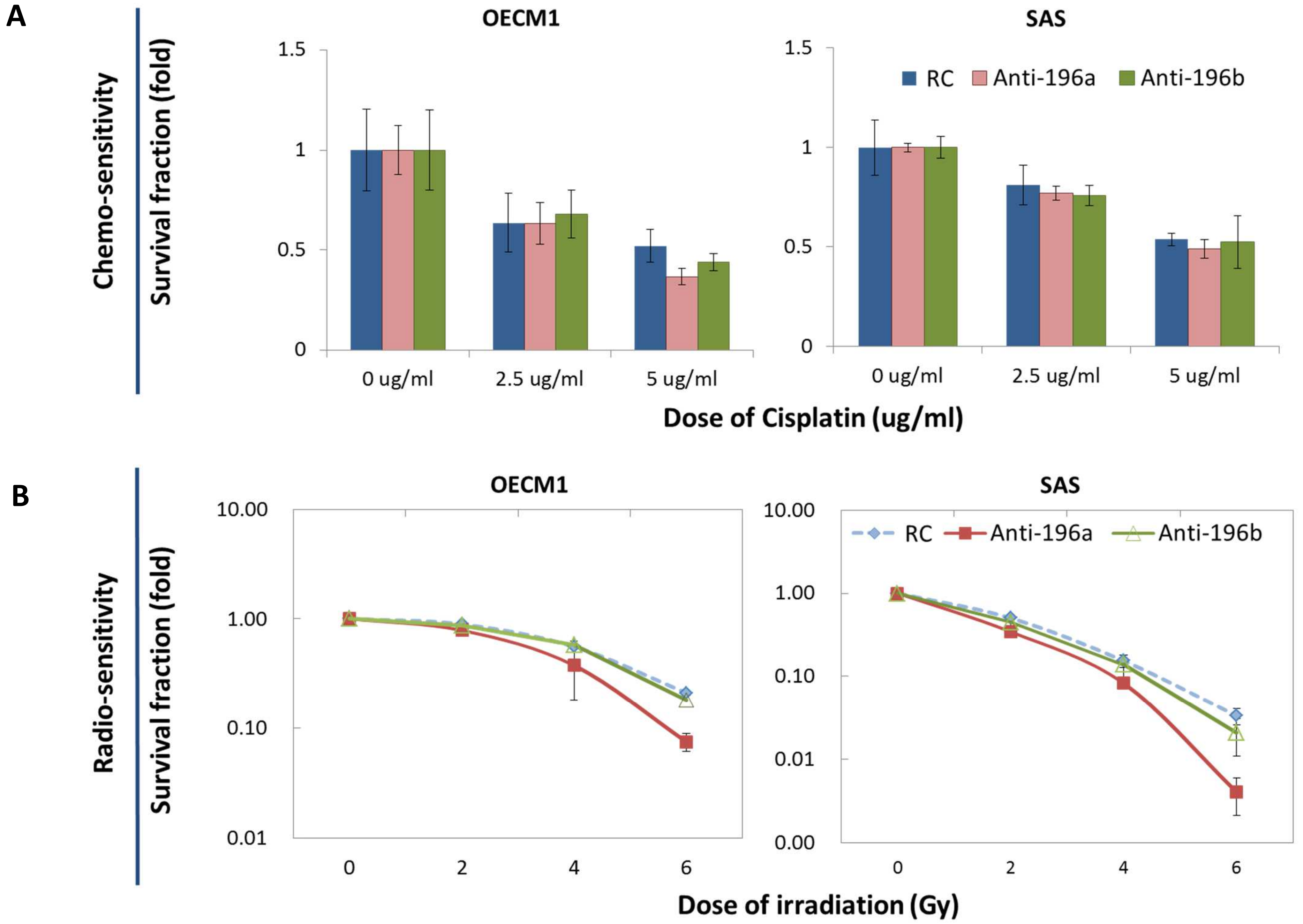

Supplementary Figure S3

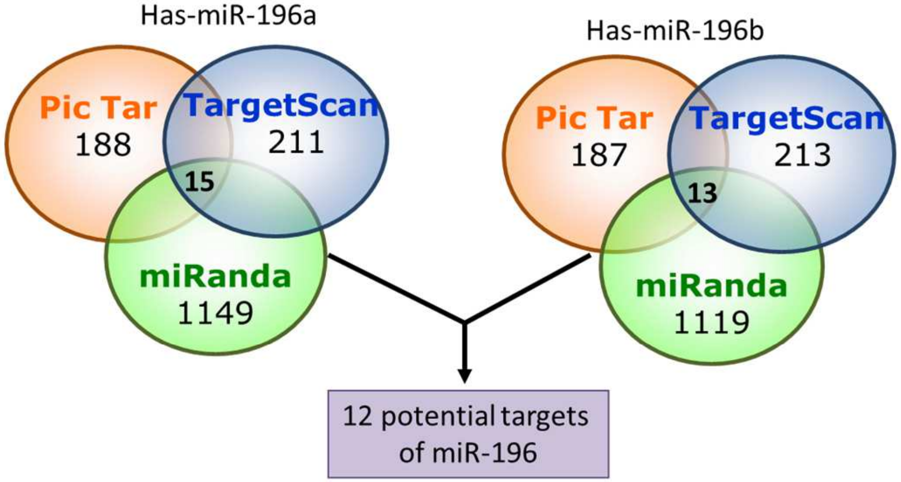

Target gene	Human RefSeq Id	CHR	Annotation
ABCB9	NM_203445	11	Homo sapiens ATP-binding cassette, sub-family B (MDR/TAP), member 9
CDYL	NM_004824	X	Homo sapiens chromodomain protein, Y-like , transcript variant 1
COL1A2	NM_000089	2	Homo sapiens collagen, type I, alpha 2
GATA6	NM_005257	19	Homo sapiens GATA binding protein 6
HAND1	NM_004821	19	Homo sapiens heart and neural crest derivatives expressed 1
HOXA5	NM_019102	16	Homo sapiens homeo box A5
HOXB6	NM_156036	1	Homo sapiens homeo box B6
HOXB7	NM_004502	8	Homo sapiens homeo box B7
HOXC8	NM_022658	9	Homo sapiens homeo box C8
MGAT4A	NM_012214	12	Homo sapiens mannosyl (alpha-1,3-)-glycoprotein beta-1,4-N-acetylglucosaminyltransferase, isoenzyme A
SMCR8	NM_144775	2	Homo sapiens Smith-Magenis syndrome chromosome region, candidate 8
NME4	NM_005009	8	Homo sapiens non-metastatic cells 4, protein expressed in

Supplementary Figure S4

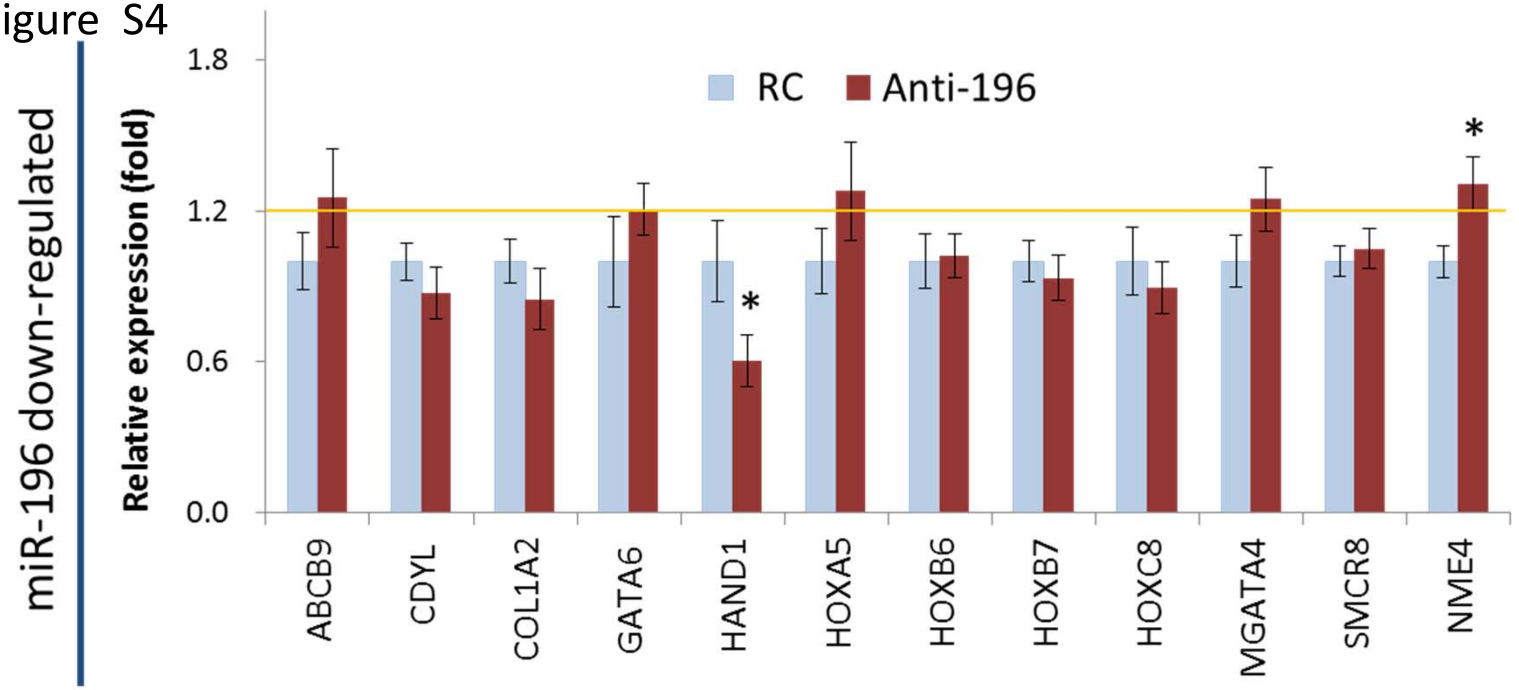

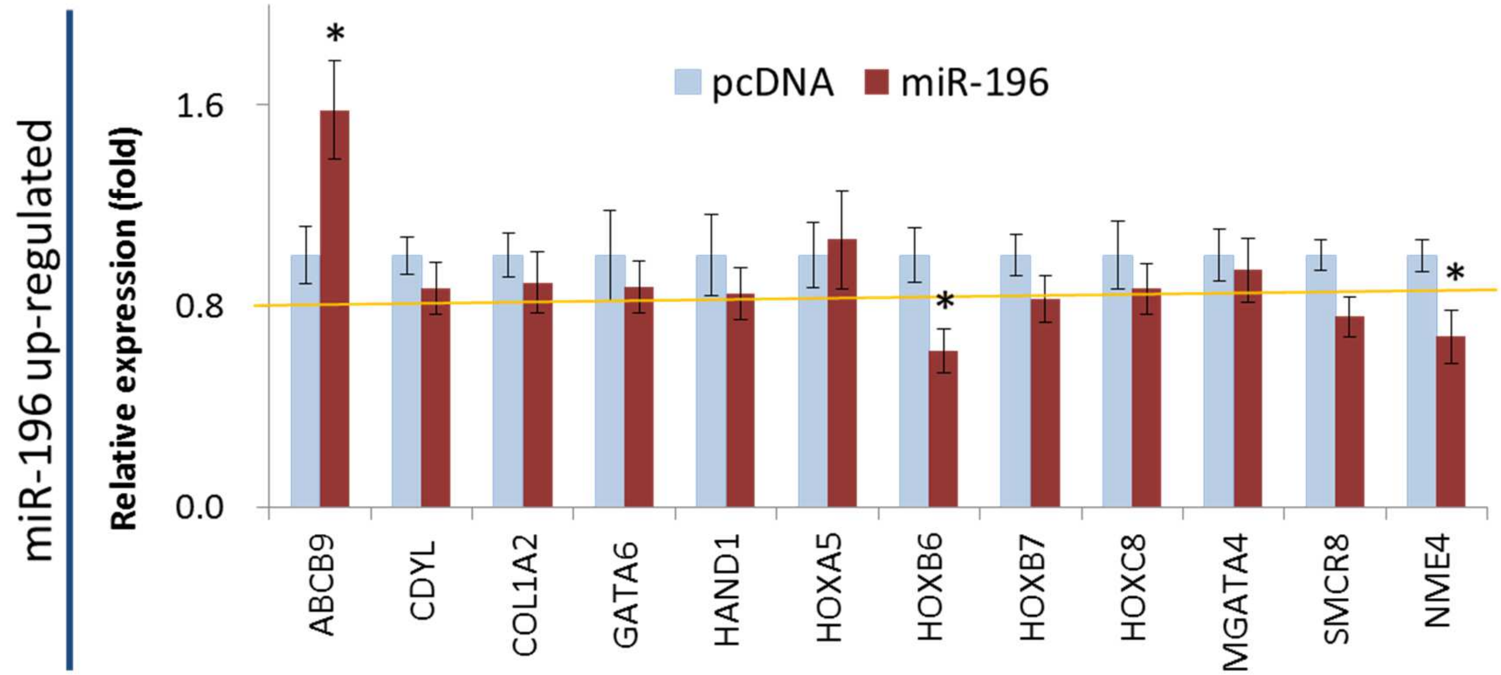

Supplementary Figure S5

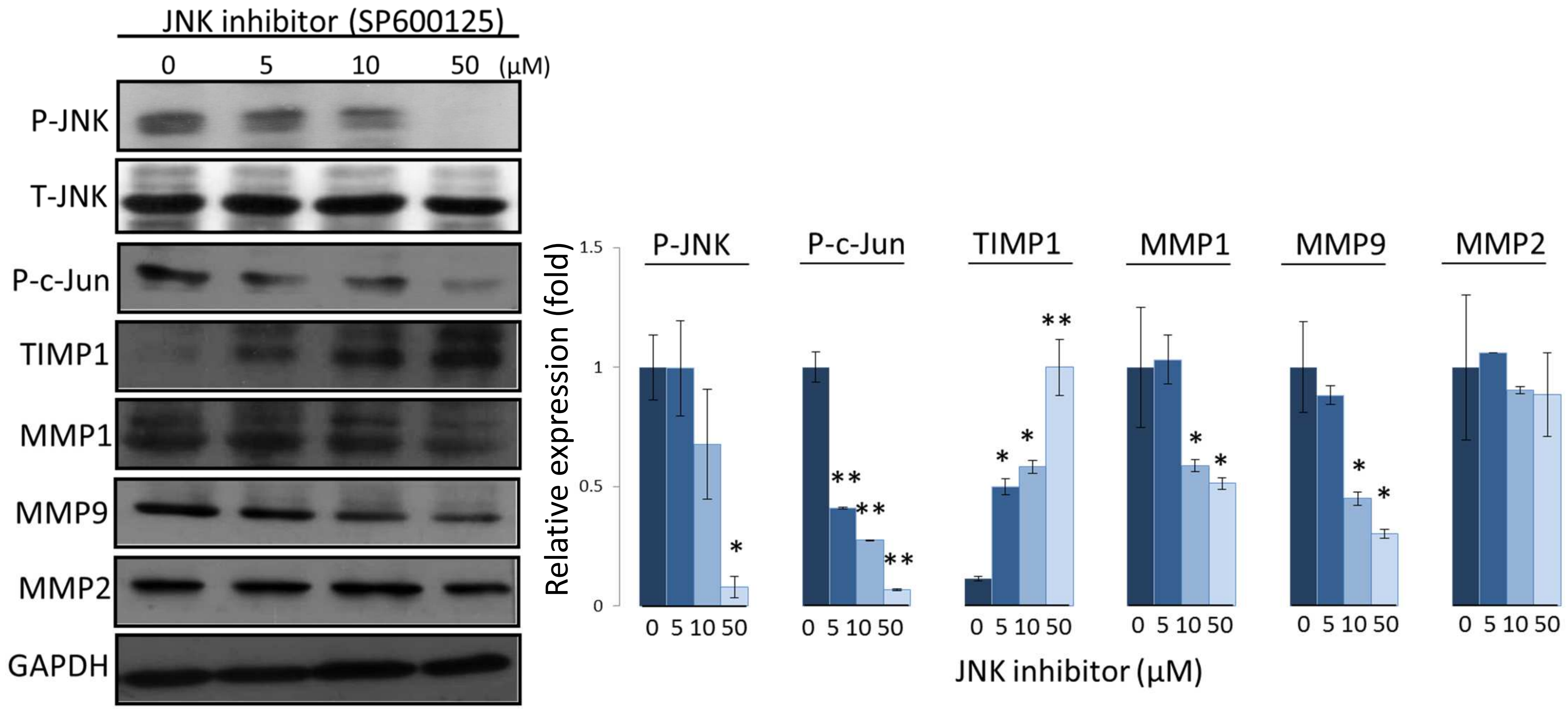

Supplementary Figure S6

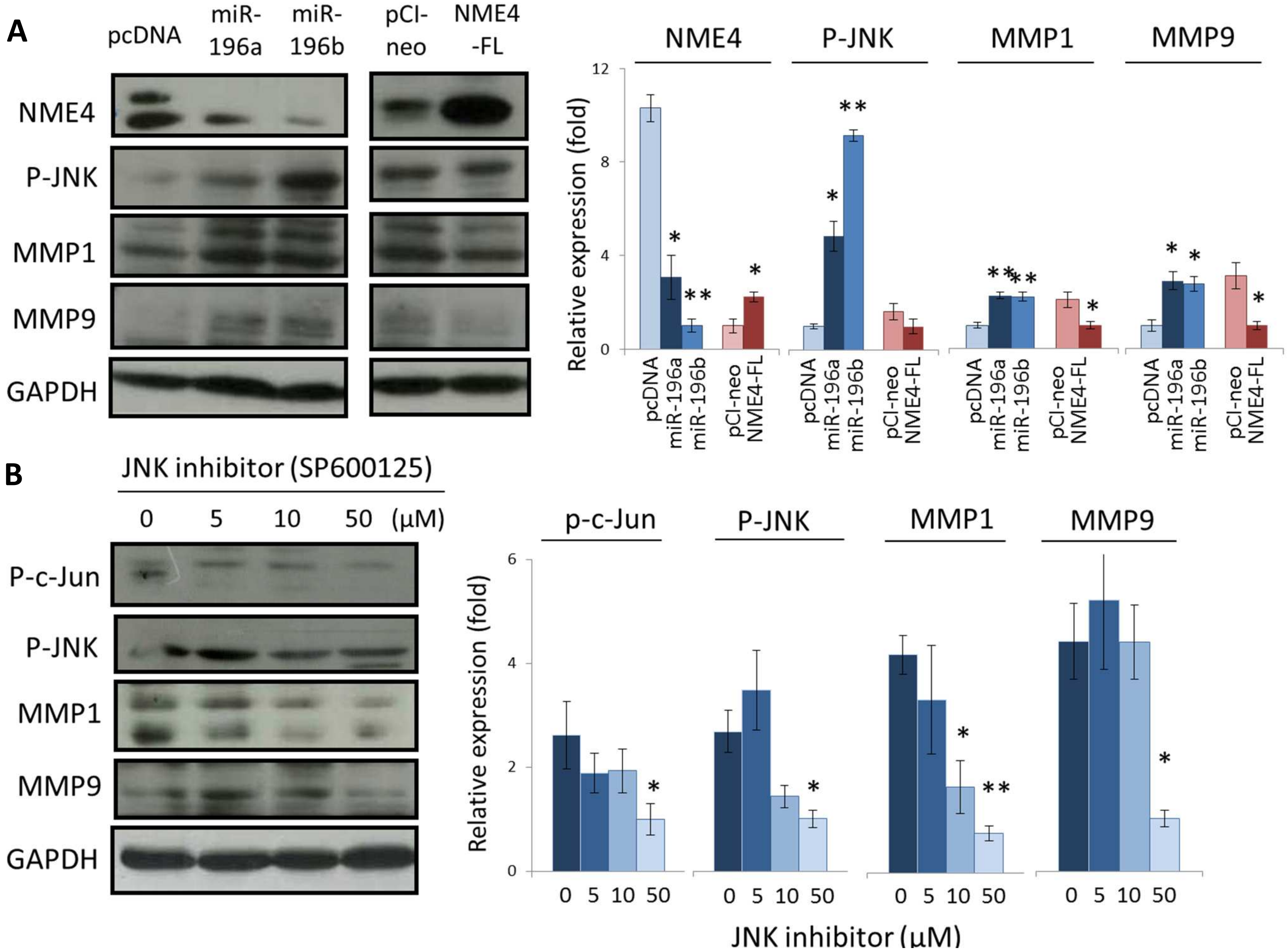

Supplement: Supplementary file 2 — Additional file 2: Figure S1: Both miR-196a and miR-196b has minimal effect on cell growth and colony formation in OECM1 and SAS cells. (A) After inhibition of miR-196s by antagomirs, cells were re-seeded to allow cell growth, and cell numbers were measured. (B) Cells were transfected with miR-196s over-expression plasmid to increase miR-196s expression. Clonogenic assay were performed and the number of colonies was determined. Figure S2. Neither miR-196a nor miR-196b affected chemosensitivity, whereas only miR-196a protects cells against irradiation (A) Chemoresistance was determined by the relative number of surviving cells after treatment with serial concentrations (0–5 μg/ml) of cisplatin for 2 days. In each sample, cell survival was counted and compared to the number of surviving untreated cell. (B) Radioresistance was determined using a clonogenic survival assay after treatment with various doses (0–6 Gy) of radiation for 7 days. Figure S3. Bioinformatic prediction of the regulatory targets of miR-196s using PicTar, TargetScan, and miRanda computational prediction software. The shared genes predicted by the three different prediction methods are listed in the table. Figure S4. Validation of the predicted genes by RT-qPCR. After transfection of the miR-196 antagomirs or overexpression plasmids into OECM1 cells, the mRNA level of each gene was determined by RT-qPCR. Relative expression was determined after each gene was normalized to the internal control (Actin). Figure S5. Effect of the JNK phosphorylation inhibitor SP600125 on TIMP1 and MMP protein expression in OECM1 cells. Protein expression was determined by western blot. Figure S6. (A) Effects of miR-196s or NME4 plasmid transfection on p-JNK, MMP1 and MMP9 in SAS cells. (B) Effect of the JNK phosphorylation inhibitor SP600125 on MMP protein expression in SAS cells. Protein expression was determined by western blot. (PDF 1 MB) [file 12943_2014_1420_MOESM2_ESM.pdf]
